# Supplementary figures and images for: Computational analysis of memory consolidation following inhibitory avoidance (IA) training in adult and infant rats: Critical roles of CaMKIIα and MeCP2
Source: PLoS Comput Biol. 2022 Jun 27;18(6):e1010239. doi: 10.1371/journal.pcbi.1010239 (PMC9269953; doi:10.1371/journal.pcbi.1010239)

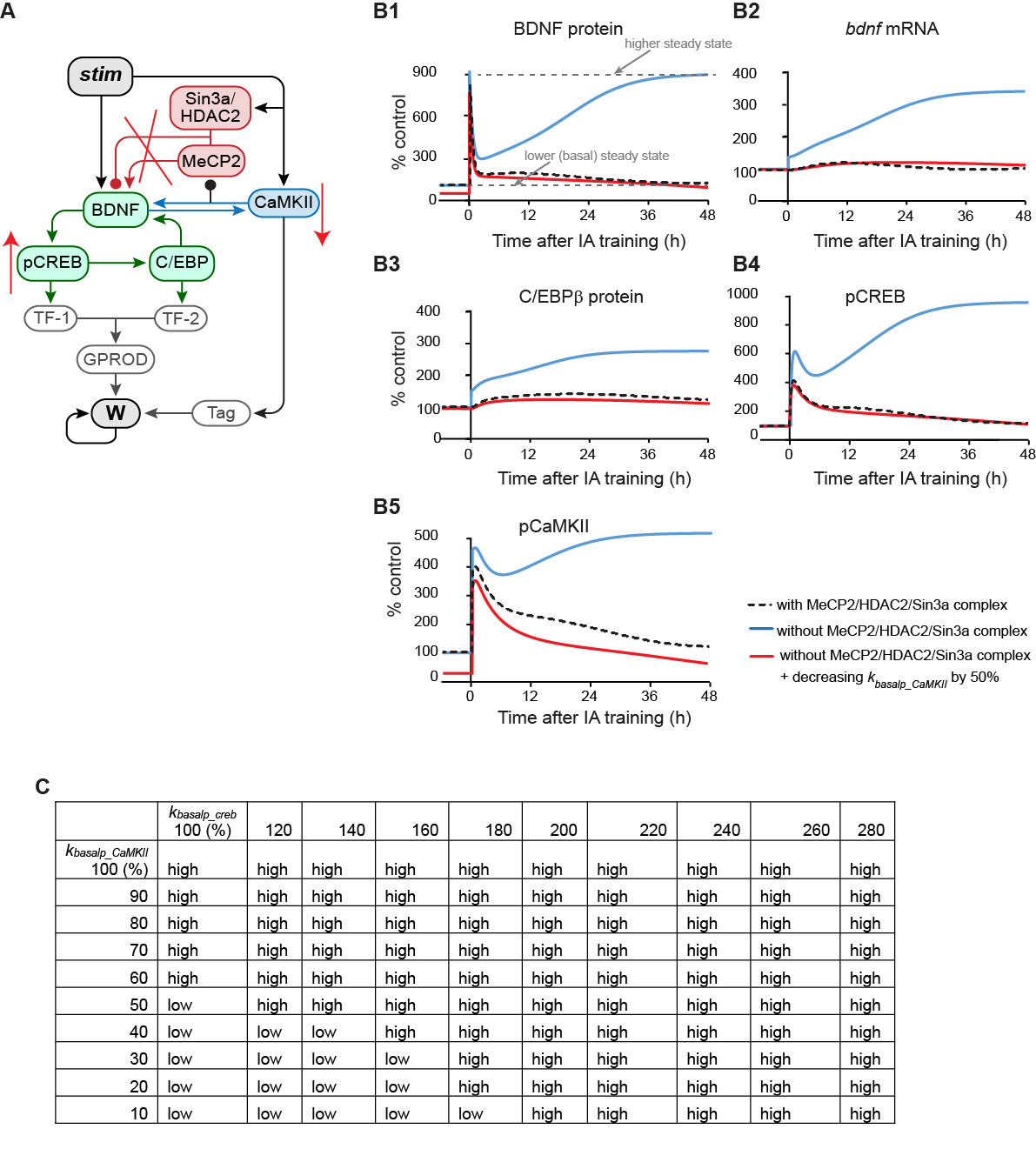

Supplement: S1 Fig — (A1) Modification of model. Increased basal phosphorylation of CREB kbasalp_creb, concurrent with decreased basal phosphorylation of CaMKIIα kbasalp_CaMKII, in the absence of the effects of MeCP2/HDAC2/Sin3a (red) ([Ecomp] = 0; [EMeCP2] = 0). (B) Example of dynamics of BDNF protein/mRNA (B1-2), C/EBPβ protein (B3), pCREB (B4), and pCaMKIIα (B5) after training in the presence (black dashed) or absence (blue) of the effects of MeCP2/HDAC2/Sin3a. In the absence of MeCP2/HDAC2/Sin3a inhibitory complex, the variables were switched to a higher steady states after training (blue). However, decreasing kbasalp_CaMKII by ~50% blocked the bistable switch (red). (C) Summary table. The steady states of variables 48 h after training with kbasalp_creb increasing from the standard value in Table 1 to ~300% of the standard value, and kbasalp_CaMKII decreasing from the standard value in Table 1 to 10% of the standard value. (TIF) [file pcbi.1010239.s001.tif]

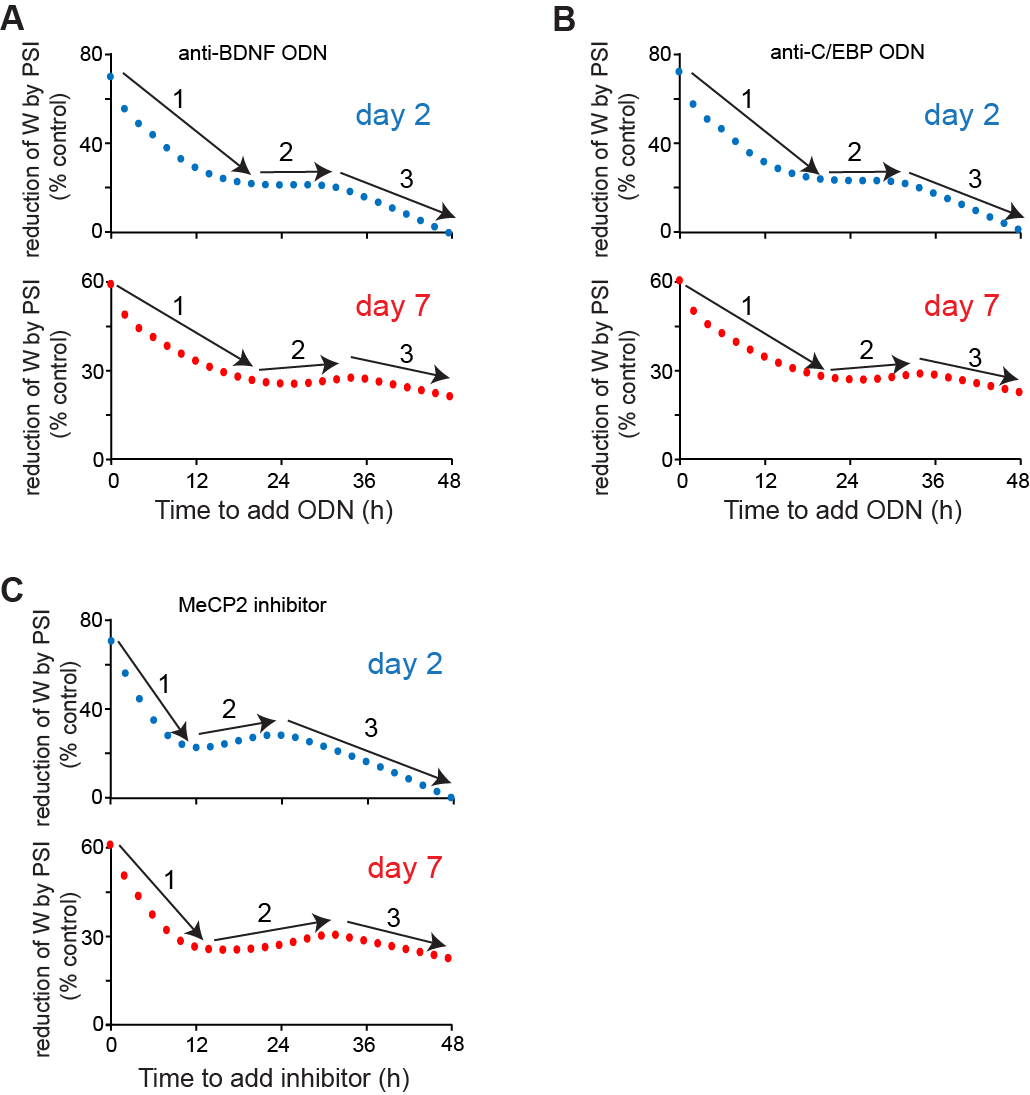

Supplement: S2 Fig — (A) Reduction of synaptic weight W at day 2 (blue curve) and 7 (red curve) after training, with the addition of anti-BDNF ODN initiated at varying times. (B) Reduction of synaptic weight W at day 2 (blue curve) and 7 (red curve) after training, with the addition of anti-C/EBP ODN initiated at varying times. (C) Reduction of synaptic weight W at day 2 (blue curve) and 7 (red curve) after training, with the addition of MeCP2 inhibitor initiated at varying times. ‘1’,’2’,’3’ represent different phases. (TIF) [file pcbi.1010239.s002.tif]
